# Etanercept Attenuates Traumatic Brain Injury in Rats by Reducing Brain TNF-**α** Contents and by Stimulating Newly Formed Neurogenesis

**DOI:** 10.1155/2013/620837

**Published:** 2013-04-21

**Authors:** Chong-Un Cheong, Ching-Ping Chang, Chien-Ming Chao, Bor-Chih Cheng, Chung-Zhing Yang, Chung-Ching Chio

**Affiliations:** ^1^Department of Intensive Care Medicine, Chi Mei Medical Center, Liouying, Tainan, Taiwan; ^2^Department of Biotechnology, Southern Taiwan University of Science and Technology, Tainan, Taiwan; ^3^Department of Surgery and Department of Intensive Care Medicine, Chi Mei Medical Center, Liouying, Tainan, Taiwan; ^4^Department of Surgery, Chi Mei Medical Center, Yung-Kang, Tainan, Taiwan; ^5^The Ph.D. Program for Cancer Biology and Drug Discovery, College of Medical Science and Technology, Taipei Medical University, Taipei 110, Taiwan

## Abstract

It remains unclear whether etanercept penetrates directly into the contused brain and improves the outcomes of TBI by attenuating brain contents of TNF-**α** and/or stimulating newly formed neurogenesis. Rats that sustained TBI are immediately treated with etanercept. Acute neurological and motor injury is assessed in all rats the day prior to and 7 days after surgery. The numbers of the colocalizations of 5-bromodeoxyuridine and doublecortin specific markers in the contused brain injury that occurred during TBI were counted by immunofluorescence staining. Enzyme immunoassay for quantitative determination of TNF-**α** or etanercept in brain tissues is also performed. Seven days after systemic administration of etanercept, levels of etanercept can be detected in the contused brain tissues. In addition, neurological and motor deficits, cerebral contusion, and increased brain TNF-**α** contents caused by TBI can be attenuated by etanercept therapy. Furthermore, the increased numbers of the colocalizations of 5-bromodeoxyuridine and doublecortin specific markers in the contused brain tissues caused by TBI can be potentiated by etanercept therapy. These findings indicate that systemically administered etanercept may penetrate directly into the contused brain tissues and may improve outcomes of TBI by reducing brain contents of TNF-**α** and by stimulating newly formed neurogenesis.

## 1. Introduction

Traumatic brain injury (TBI) caused by a direct mechanical insult to the brain induces cerebral contusion and motor and cognitive dysfunction [1–4]. Recent studies have indicated that populations of damaged or destroyed neurons can be replenished by proliferation of neural stem cells (NSCs) [5, 6] or newly forming neurons [4]. NSCs have the potential to differentiate into neural phenotypes.

Doublecortin (DCX), a microtubule-associated protein, is specifically expressed in all migrating neuronal precursors of the developing brain [7, 8]. DCX expression is retained mainly within areas of the subventricular zone (SVZ) of the lateral ventricles and the subgranular zone (SGZ) at the dentate gyrus/hilus interface of hippocampus [8, 9] in the adult brain. Following an insult to the brain, the increased number of divided cells in the SVZ and SGZ is newly formed immature neurons and expresses DCX [1, 7, 10]. Because of its association with neurogenic processes, DCX expression levels in the adult brain reflect neurogenesis [7].

Inhibiting tumor necrosis factor-alpha (TNF-*α*) with etanercept is effective for attenuating TBI-induced cerebral contusion, motor and cognitive dysfunction, astrocytic and microglial activation, and activated inflammation [11, 12]. However, it is not known whether systemically administered etanercept penetrates injured brain tissue and attenuates the TBI-induced brain dysfunction by stimulating DCX-associated neurogenesis. We hypothesize that it does. Therefore, we examine the relationship between DCX-associated neurogenesis and the TBI-induced formation of cerebral contusions around a damaged brain area. Changes in 5-bromodeoxyuridine-(BrdU-) DCX double expression are analyzed using immunohistochemistry to investigate the relationship between DCX-associated neurogenesis and the formation of cerebral contusions and cognitive dysfunction in rats treated with and without etanercept. Quantitative enzyme immunoassays are used to analyze etanercept levels in the brain after its systemic administration to ascertain whether it had passed through the blood-brain barrier (BBB) during TBI.

## 2. Materials and Methods

### 2.1. Animals

Male Sprague-Dawley rats (weight, 254 ± 10 g) were purchased from the National Animal Laboratory Center of the National Science Council (Taipei, Taiwan). Four rats were housed together at an ambient temperature of 22 ± 1°C with a 12 h light-dark cycle. Pelleted rat chow and tap water were available *ad libitum*. All protocols were approved by the Animal Ethics Committee of the Chi Mei Medical Center. We did as much as possible to minimize the rats' discomfort during surgery and in the recovery period.

### 2.2. Surgery

The rats were intraperitoneally (i.p.) anesthetized with sodium pentobarbital (25 mg/kg) (Sigma-Aldrich, St. Louis, MO, USA) and intramuscularly (i.m.) with a mixture containing ketamine (4.4 mg/kg) (Nang Kuang Pharmaceutical, Tainan, Taiwan), atropine (0.02633 mg/kg) (Sintong Chemical, Taoyuan, Taiwan), and xylazine (6.77 mg/kg) (Bayer, Leverkusen, Germany). Each rat was placed in a stereotaxic frame, and its scalp was sagittally incised. The rats were then subjected to a lateral fluid percussion injury [13]. After the scalp had been incised, a 4.8 mm circular craniotomy was done midway between lambda and bregma, 3.0 mm to the right of the central suture. A modified Luer-lock connector (trauma cannula) with an inner diameter of 2.6 mm was secured in the craniotomy with cyanoacrylate adhesive and dental acrylic. A moderate percussion (2.2 atm) was produced by rapidly injecting a small volume of saline into the closed cranial cavity with a fluid percussion device (VCU Biochemical Engineering, Richmond, VA, USA). The rat was removed from the device, the acrylic was removed, and the incision was sutured. Each injured and sham-injured animal for the percussion model was closely evaluated immediately after percussion for behavioral recovery.

### 2.3. Experimental Groups

The rats were randomly allocated into one of three groups: (i) TBI + vehicle (TBI + V): the rats were subjected to TBI and injected with normal saline (1 mL/kg; i.p.) once every 12 h for 3 consecutive days (*n* = 8); (ii) TBI + etanercept (TBI + E): the rats were subjected to TBI and injected with etanercept (0.1 mg/kg; i.p.) once every 12 h for 3 consecutive days (*n* = 8); and (iii) sham-TBI: the rats were subjected to the same surgical procedures as described in Section 2.2 but not to percussion-induced TBI (*n* = 8).

### 2.4. Experimental Procedures

In Experiment 1, etanercept (Enbrel; Wyeth Pharmaceuticals, New Lane, Havant, Hampshire, UK) or saline was injected immediately after TBI once every 12 h for 3 consecutive days, and the effect on the maximal angle of an inclined plane that the rats could cling to, as well as neurological severity score (NSS), was assessed 7 days after TBI.

In Experiment 2, etanercept or saline was randomly administered immediately after TBI once every 12 h for 3 consecutive days, and the effect on the rats' cerebral contusion zone was assessed 7 days after TBI.

In Experiment 3, etanercept or saline was randomly administered immediately after TBI once every 12 h for 3 consecutive days, and the effect on double-immunofluorescence staining for doublecortin (DCX) and neuronal nuclei (NeuN) in the rats' damaged brain areas was assessed 7 days after TBI.

In Experiment 4, etanercept or saline was injected immediately after TBI once every 12 h for 3 consecutive days, and the effect on the enzyme immunoassay for the quantitative determination of etanercept in the rats' damaged brain tissue was assessed 7 days after TBI.

### 2.5. Neurological Function Evaluation

Acute neurological injury was assessed in all rats the day prior to and 7 days after surgery using an NSS [14]. NSS is a composite of the motor, sensory, and reflex tests. One point was given for failure to perform a task. Thus, the higher the score is, the more severe the injury is, with a maximum of 14 points.

The inclined plane was used to measure limb strength [15]. The rat was placed, facing right and then left, perpendicular to the slope of a 20 × 20 cm ruffed surface of an inclined plane starting at an angle of 55°. The angle was increased or decreased in 5° increments to determine the maximal angle at which a rat could hold to the plane. The data for each day were the mean of the left- and right-side maximal angles. All behavioral tests were examined and independently scored by two observers who were unaware of what treatment the rats had been given. These scores were averaged to arrive at one score for each rat for the behavioral session. The tests were tested before injury and on day 7 after etanercept treatment.

### 2.6. Tumor Necrosis Factor-*α* (TNF-*α*) Contents of Ischemic Cerebral Homogenate

Cerebral hemispheres were quickly dissected free and kept on ice in physiological salt solution containing 5 mM glucose. Segments of cerebral cortex (75 mg, i.e., approximately the weight of each cerebral hemisphere) were weighted, cut into small pieces, dispersed by aspiration into a pipette, and suspended in 1 mL of physiological salt solution in a test tube. Samples were kept on wet ice for 20 min. The supernatants were used for measuring TNF-*α* concentrations. TNF-*α* concentrations were measured using commercial enzyme-linked immunosorbent assay (ELISA) kits (Biosource International Inc. Boshide Company, Wuhan, China) and following the manufacturer's instructions. The minimum detectable concentrations of TNF-*α* were 1.1 pg/mL. There was no cross-reactivity reported with other cytokines. All samples were assayed in duplicate.

### 2.7. Cerebral Contusion Assay

The triphenyltetrazolium (TTC) staining procedures followed those described elsewhere [16]. Four days after TBI, all the rats were deeply anesthetized (sodium pentobarbital, 100 mg/kg; i.p.) and then intracardially perfused with saline. Their brain tissue was then removed, immersed in cold saline for 5 min, and sliced into 2.0 mm sections with a tissue slicer. The brain slices were incubated in 2% TTC dissolved in phosphate buffer saline (PBS) for 30 min at 37°C and then transferred to 5% formaldehyde solution for fixation. The volume of contusion, as revealed by negative TTC stains indicating dehydrogenase-deficient tissue, was measured in each slice and summed using computerized planimetry (Image-Pro Plus 5.0; Media Cybernetics, Bethesda, MD, USA). The volume of contusion was calculated as 2 mm (thickness of the slice) × (sum of the contusion area in all brain slices (mm^2^)).

### 2.8. Immunohistochemistry

To evaluate the proliferation of cells, the rats were injected once daily for 3 consecutive days after TBI with 5-bromodeoxyuridine (BrdU) (50 mg/kg; i.p.) (Roche Diagnostics, Indianapolis, IN, USA) dissolved in PBS. The rats were killed and then perfused intracardially with 300 mL of 0.1 M PBS (pH 7.4-7.5), followed by 300 mL 4% paraformaldehyde (PFA) in PBS (pH 7.4-7.5) 7 days after TBI. Their brains were removed and stored in PFA for 3 days and then were sliced into serial coronal sections (50 *μ*m thick) and the maximum size of the lesion using a microslicer (Dousaka EM, Kyoto, Japan). The section was used for DCX immunostaining, followed by counterstaining with double-immunofluorescence staining for DCX and BrdU.

Serial 50 *μ*m sections corresponding to coronal coordinates 0.8 mm to 5.3 mm posterior to the bregma were incubated in 2 mol/L HCL for 30 min, rinsed in 0.1 mol/L boric acid (pH8.5) for 3 min at room temperature, and then incubated with primary antibodies in phosphate-buffered saline (PBS) containing 0.5% normal bovine serum at 4°C overnight; secondary antibodies were incubated for 1 h at room temperature. The antibodies therein were, sequentially, rabbit anti-DCX antibody (Cell Signaling Technology, 1 : 200), rat anti-BrdU antibody (Abcam, 1 : 200), goat anti-rabbit IgG-H&L antibody (Abcam, 1 : 400), and goat anti-rat IgG antibody (Abcam, 1 : 400). The sections were overslipped with the mounting medium (Fluorescent Mounting Medium; Dako). The labeled cells were calculated in 5 coronal sections from each rat and expressed as the mean number of cells per section. For negative control sections, all the procedures were without the primary antibody. Primary and secondary antibodies for multiple staining are listed in Table 1.

### 2.9. Enzyme Immunoassay for Quantitative Determination of Etanercept in Brain Tissue Samples

An enzyme immunoassay (Etanercept (Enbrel) ELISA Q-ETA; Matriks Biotek Laboratories, Ankara, Turkey), a solid phase enzyme-linked immunosorbent assay (ELISA) based on the sandwich principle, was used for the quantitative determination of etanercept in brain tissue samples. Standards and samples were incubated in a microtiter plate coated with the monoclonal antibody specific for etanercept. After the incubation, the wells were washed. A biotin-labeled tracer monoclonal antibody against etanercept was added and bound to etanercept captured by the first monoclonal antibody on the surface of the wells. After a second incubation, the wells were washed and then streptavidin-horse radish peroxidase (HRP) was added and bound to the biotin-conjugated probe. After a third incubation, the wells were washed and the bound enzymatic activity was detected by adding chromogen substrate. The color developed is proportional to the amount of etanercept in the sample or standard. Results of the sample were directly determined using the standard curve.

### 2.10. Bromodeoxyuridine Labeling

Bromodeoxyuridine (BrdU), a thymidine analogy that is incorporated into the DNA of dividing cells during S-phase, was used for mitotic labeling (Roche Diagnostics, Indianapolis, USA; 50 mg/kg). The labeling protocol has been described previously [17]. BrdU was administered intraperitoneally daily for 3 consecutive days after FPI. The FPI animals were killed 7 days after FPI for BrdU labeling. The BrdU immunostaining procedure with specific antibody against BrdU (1 : 400; Roche Diagnostics) and quantification of BrdU-immunoreactive cells have been described previously [17].

### 2.11. Statistical Analysis

The data are mean ± standard deviation (SD). Statistical analysis was done using one-way analysis of variance (ANOVA) with Fisher's post hoc test. Analyses for behavioral variables used Student's unpaired *t*-test to compare variables between groups. Bonferroni's analysis was then performed when appropriate, to determine posthoc significance at individual time point. Data was analyzed using Statiatica Software, and, in all cases, statistical significance was set at *P* < 0.05.

## 3. Results

### 3.1. TBI Caused Neurological and Motor Dysfunction, Which Etanercept Attenuated

**Figure 1:**
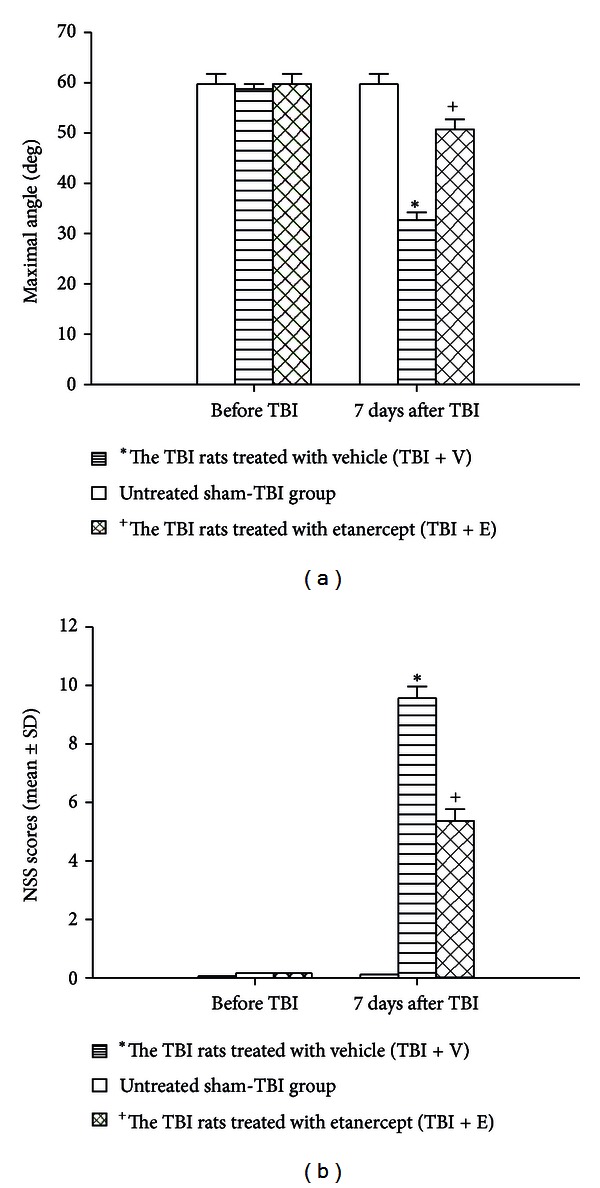
Etanercept attenuated TBI-induced increased neurological NSS and decreased motor performance. *The TBI rats treated with vehicle (TBI + V) (lined column; *n* = 8) showed a significant increase in NSS (*P* < 0.01) and a significant decrease in maximal angle (*P* < 0.05) compared with the untreated sham-TBI group (white column) 7 days after TBI. ^+^The TBI rats treated with etanercept (TBI + E) (crossed column; *n* = 8) showed a significant decrease in NSS (*P* < 0.05) and a significant increase in maximal angle (*P* < 0.05) compared with the (TBI + V) group (lined column; *n* = 8).

Seven days after the rats had been subjected to TBI, behavioral tests revealed that the NSS scores of both the (TBI + V) group and the (TBI + E) group were significantly (*P* < 0.05) higher than those of the untreated sham-TBI group (Figure 1). However, compared with those of the (TBI + V) group, the NSS scores values of the (TBI + E) group were significantly (*P* < 0.05) lower. In contrast, motor function tests showed that the maximal angles of the (TBI + V) group were significantly lower than those of the sham-TBI group (Figure 1). Compared with those of the (TBI + V) group, the maximal degrees were significantly (*P* < 0.05) higher in the (TBI + E) group.

### 3.2. TBI Caused Cerebral Contusion and Increased Cerebral Levels of TNF-*α*, Which Etanercept Attenuated

**Figure 2:**
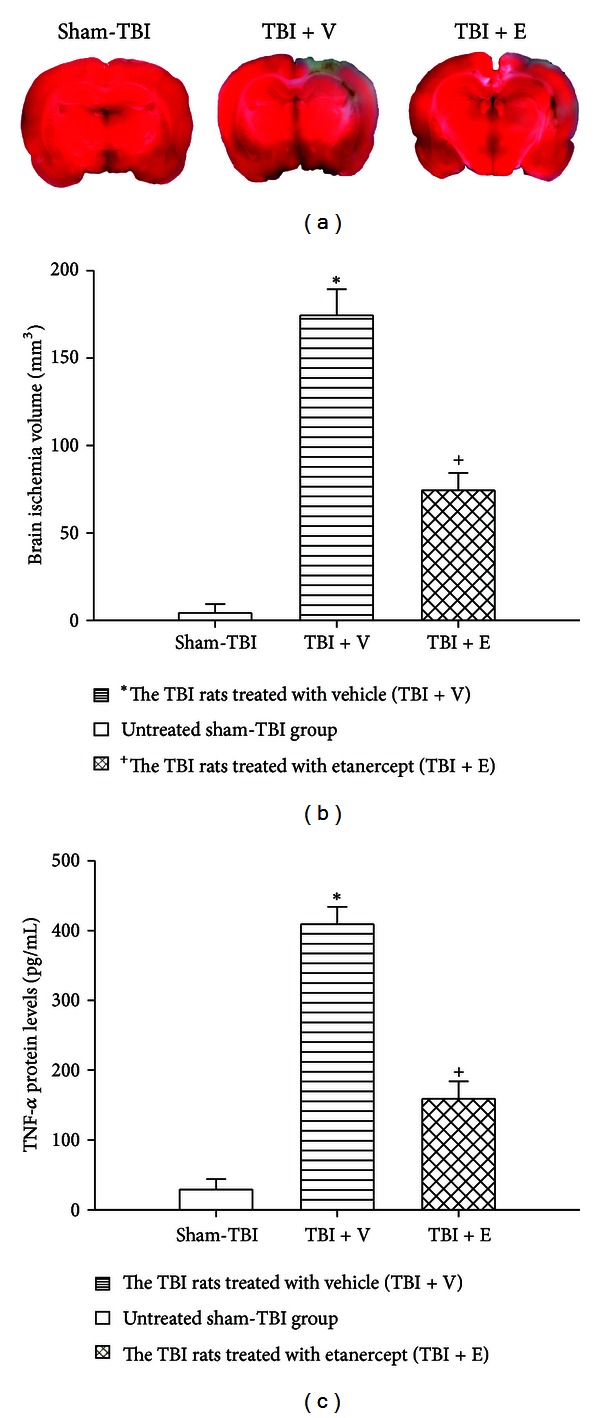
Etanercept attenuated TBI-induced increased brain contused volume and increased brain TNF-*α* contents. *The TBI rats treated with vehicle (TBI + V) (lined column; *n* = 8) showed a significant increase in both brain contused volume and TNF-*α* contents (*P* < 0.01) compared with the untreated sham-TBI group (white column) 7 days after TBI. ^+^The TBI rats treated with etanercept (TBI + E) (crossed column; *n* = 8) showed a significant decrease in both brain contused volume and brain TNF-*α* contents (*P* < 0.05) compared with the (TBI + V) group (lined column; *n* = 8). Top panels depict representative contused stainings for one sham TBI rat, one TBI + V rat, and one TBI + E rat.

Seven days after the rats had been subjected to TBI, TTC staining and ELISA kits showed that the (TBI + V) group had significantly (*P* < 0.01) larger areas of brain contusion and larger contents (pg/mL) of brain TNF-*α* than did the sham-TBI group (Figure 2). Both the cerebral contusion and the increased brain TNF-*α* contents (pg/mL) were significantly (*P* < 0.01) smaller in the (TBI + E) group than in the (TBI + V) group (Figure 2).

### 3.3. TBI Increased the Numbers of DCX-BrdU Double-Positive Cells around the Damaged Brain Areas, Which Etanercept Enhanced

**Figure 3:**
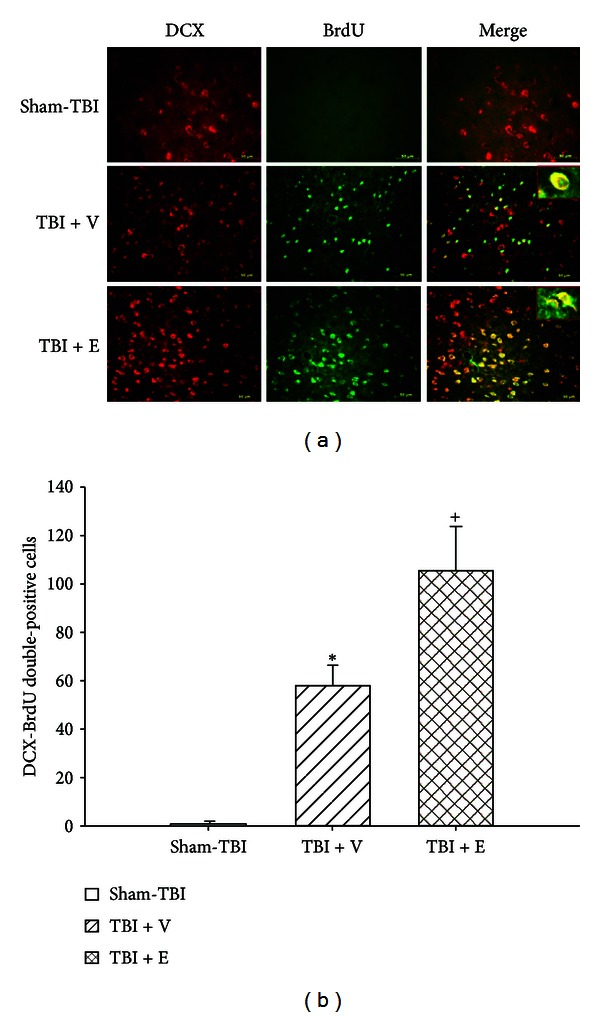
Mean ± standard deviation values of the number of BrdU-DCX-positive cells in the core ischemic cortex. Sham-TBI (white column): rats given a sham traumatic brain injury (TBI) operation; TBI + V (lined column): TBI rats treated with vehicle solution; TBI + E (crossed column): TBI rats treated with etanercept solution. The data were obtained 7 days after TBI or sham-TBI operation (*n* = 8). *P < 0.05 compared with the sham-TBI group; ^+^ *P* < 0.05 compared with the TBI + V group. Right panels depict representative DCX-BrdU double stainings for one sham-TBI rat, one TBI + V rat, and one TBI + E rat.

**Figure 4:**
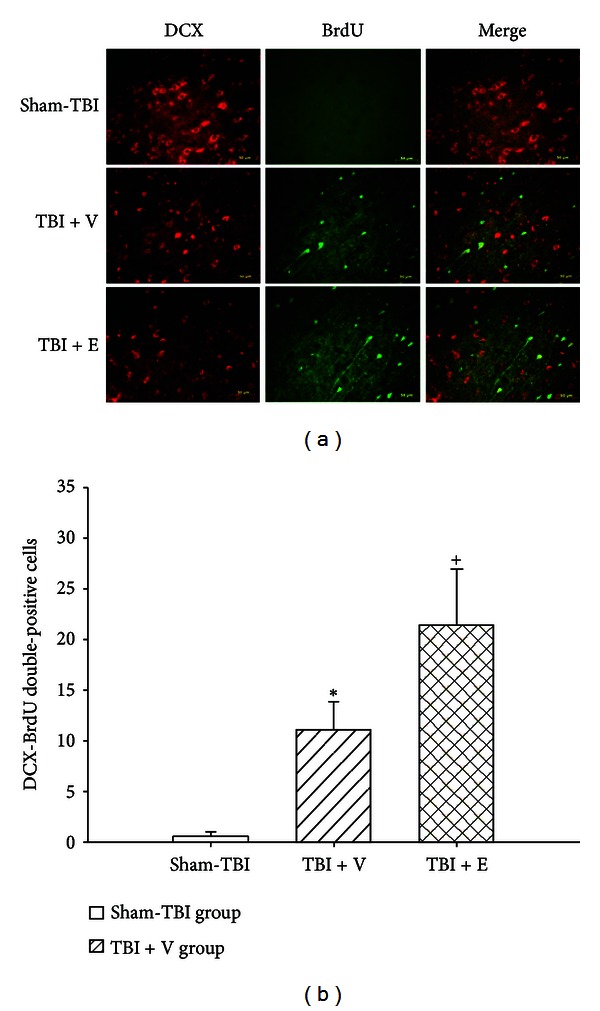
Mean ± standard deviation values of the number of BrdU-DCX-positive cells in the penumbra ischemic cortex (see the legend of Figure 1 for group abbreviations). The data were obtained 7 days after TBI or sham-TBI operation (*n* = 8). *P < 0.05 compared with the sham-TBI group (white column); ^+^ *P* < 0.05 compared with the TBI + V group (lined column). Right panels depict representative DCX-BrdU double stainings for one sham-TBI rat, one TBI + V rat, and one TBI + E rat.

**Figure 5:**
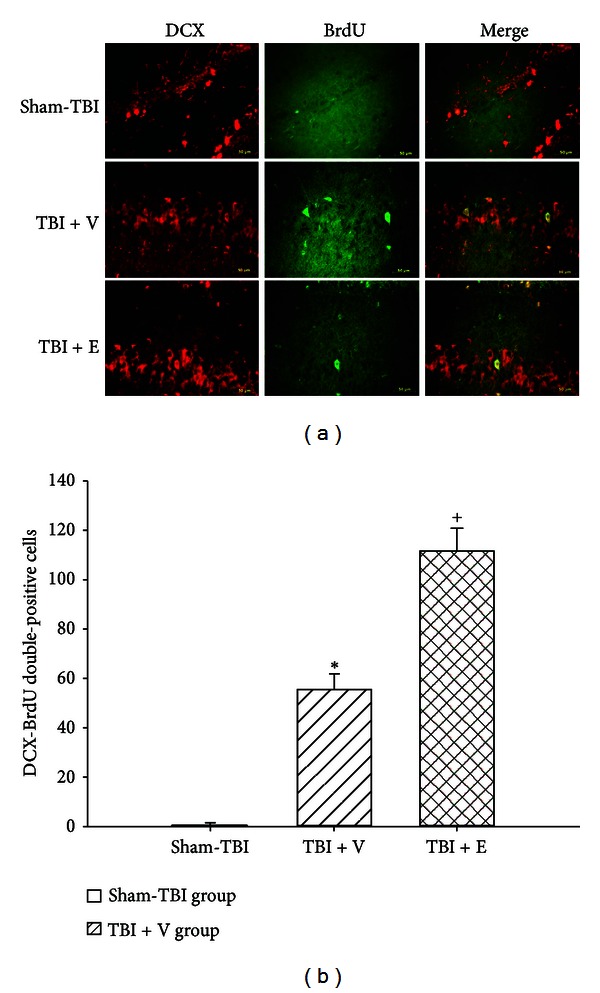
Mean ± standard deviation values of the number of BrdU-DCX-positive cells in the ischemic hippocampus. (See the legend of Figure 1 for group abbreviations). The data were obtained 7 days after TBI or sham-TBI operation (*n* = 8). *P < 0.05 compared with the sham-TBI group (white column); ^+^ *P* < 0.05 compared with the TBI + V group (lined coumn). Right panels depict representative DCX-BrdU double stainings for one sham + E rat, one TBI + V rat, and one TBI + E rat.

Seven days after the rats had been subjected to TBI, double-immunofluorescence staining showed that the cell numbers of colocalizations of both DCX and BrdU around the contused frontal cortex core (Figure 3), contused cortex penumbral (Figure 4), and contused hippocampal area (Figure 5) were significantly (*P* < 0.01) higher than those of the sham-TBI group. Additionally, the numbers of colocalizations of both DCX and BrdU around the contused cortical core, cortical penumbra, and hippocampus in the (TBI + E) group were significantly (*P* < 0.05) higher than those of the (TBI + V) group (Figures 3–5).

### 3.4. Etanercept, When Administered Systemically, Could Be Detected in the (TBI + E) Group

**Figure 6:**
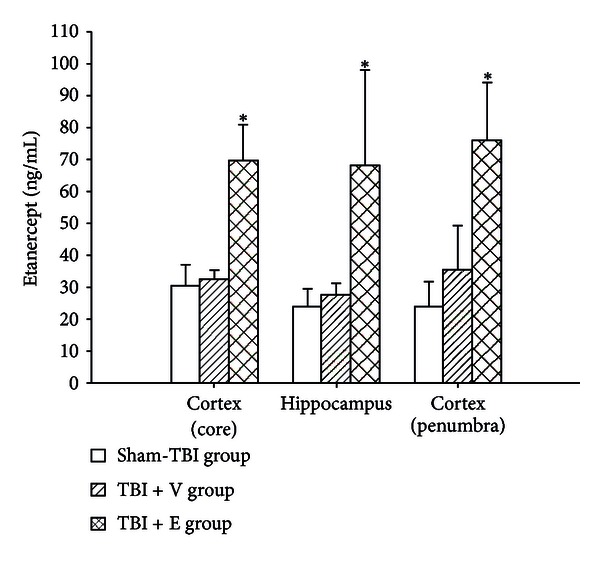
Mean ± standard deviation values of the concentrations of etanercept detected in the ischemic brain regions for the sham-TBI group (white column), the TBI + V group (lined column), and the TBI + E group (crossed column). (See the legend of Figure 1 for group abbreviations). The data were obtained 7 days after TBI or sham-TBI operation (*n* = 8). *P < 0.05 compared the TBI + V group or the sham-TBI group.

Seven days after the rats had been subjected to TBI, a quantitative enzyme immunoassay showed the levels of etanercept in the contused frontal cortex (core), hippocampus, and frontal cortex (penumbra) could be detected in the (TBI + E) group but not in the (TBI + V) group or the sham-TBI group (Figure 6).

## 4. Discussion

It has previously reported that proliferation increases three- to sixfold beginning as early as 2 days after injury, peaks during the first week after injury, and returns to baseline levels of proliferation in the dentate gyrus by 35 days [2, 10, 18, 19]. In addition, it was found that in the first week after TBI, reduced numbers of DCX-positive cells were seen in the hippocampus; a return to control levels occurred at 14 days [20]. Based on these observations, the estimation day was chosen to be 7 days after injury especially in this study, instead of 4 days after TBI as previously shown in our literature [12]. Here, we have shown that peripheral administration of etanercept is able to penetrate into the damaged frontal cortex and hippocampus to decrease novo local TNF-*α* expression, to increase numbers of the cells with the colocalization of both DCX and BrdU specific markers, and to improve neurological and motor dysfunction in rats during the first week after injury. Etanercept is both hydrophilic and of high molecular weight, and so it is prevented from crossing the intact BBB [21] in normal condition. However, we do observe that the levels of etanercept in the damaged frontal cortex and hippocampus are scientifically increased 7 days after systemic administration of etanercept. This indicates strongly that the blood-brain barrier may be brokendown during injury. In fact, the central effects of etanercept are supported by several previous reports. For example, perispinal administration of etanercept induced rapid cognitive improvement in a patient with late-onset Alzheimer's disease [22]; systemic administration of etanercept inhibited interleukin-1*β*-mediated the depression of open-field activity and reduced glucose consumption [23]; direct injection of a selective tumor necrosis factor-*α* antagonist-soluble tumor necrosis factor-*α* receptor fusion protein 15 min before and 1 h after TBI improved performance in a series of standard tasks after injury [24]; and systemic administration of etanercept attenuated spinal cord injury in rats [25]. As shown in Figure 6, the levels of etanercept given 7 days before sacrifice are so similar or identical in all structures (core, penumbra, and hippocampus). This indicates that this drug (etanercept) may accumulate in both the contused and the healthy brain tissues.

Established studies [5, 6] suggest that the mammalian nervous system has the potential to replenish populations of damaged neurons by the proliferation of neural stem cells (NSCs). The presence of NSCs has been confirmed in two portions in the adult rodent brain: one is the SVZ of the lateral cerebral ventricles [26] and the other is the SGZ at the dentate gyrus/hilus interface [27, 28]. Indeed, as shown in the present study, a direct mechanical insult to the brain 7 days after TBI induced neurogenesis, which was evidenced by an increase in DCX-BrdU double-positive cells in the damaged areas of the hippocampus, the periventricular areas, and the cortex. According to Itoh et al. [3], DCX-positive cells were present near and among the glial scars after TBI, and these cells changed from immature to mature neurons. It is believed that promoting the maturation and differentiation of newly formed immature neurons near glial scars after TBI attenuates after TBI glial-scar-induced brain dysfunction. Indeed, we showed that the number of the newly formed BrdU-DCX double-positive cells in injured brain areas (including both the core of the infarct and the hippocampus) was significantly higher in rats that had undergone TBI. The great similarity between the number of DCX-BrdU positive cells at the core of the infarct and the hippocampus suggests that newly formed cells have already migrated to the core of infarction from the hippocampus. This is consistent with findings in humans. For example, Chiaretti et al. [29] reported that early DCX concentrations in the cerebrospinal fluid (CSF) were correlated significantly with the severity of heat injury in children. In contrast, our data were not supported by those of Rola et al. [20], who found that, during the first week after TBI, there were fewer DCX-positive cells at all the time points except 48 h after injury, when there was a transient increase in mice that had underwent TBI.

Some evidence indicates that TNF-*α* plays a neuroprotective role following TBI [30, 31], whereas other evidence shows that TNF-*α* plays an important role in the pathophysiology of TBI [32–34]. Our present data demonstrate that TNF-*α* overexpression is associated with the pathological effects as well as neurological motor deficits during injury. On the other hand, at recovery process, TNF-*α* contributes to neuroanatomical plasticity as well as an improvement of locomotor activity following TBI [35]. Putting these observations together, we can derive that etanercept may improve outcomes of TBI in rats by reducing overproduction of brain TNF-*α* at least during the early stage (e.g., 7 days).

Although our present results tend to support the central effects of etanercept administered systemically, etanercept may work by blocking peripheral TNF-*α*, which, we now know, is produced by the liver as part of the peripheral response to acute central nervous system inflammation [11] or TBI [12]. Our data further indicate that peripheral administration of etanercept can be used to induce neurogenesis in nonneurogenic regions by stimulating local NSCs or recruiting NSCs from neurogenic areas to other areas of the brain during TBI.

Activated microglia are a good indicator of brain inflammation [36]. It has been documented that uncontrolled inflammation is associated with activation of microglia as well as detrimental to neurogenesis partially through production of TNF-*α* [37, 38]. This is confirmed by the present results showing about 10-fold increase in TNF-*α* at day 7 after TBI (Figure 2). The negative effect on neurogenesis by activated microglia is because of the overproduction of TNF-*α* and other mediators [39]. The contention is supported by our present data at least in part. Our present results show that suppression of production of brain TNF-*α* with etanercept significantly stimulates neurogenesis but significantly attenuates the neurological and motor deficits as well as the brain contusion during TBI in rats.

## 5. Conclusion

The current study demonstrates that TBI, in addition to inducing cerebral contusion and neurological motor deficits, induces the overproduction of TNF-*α* as well as the increased numbers of the colocalizations of BrdU and DCX specific markers in the contused bran tissues. Levels of etanercept can be detected in brain following systemic delivery of etanercept to TBI animals. In addition, cerebral contusion, neurological motor deficits, and increased brain contents of TNF-*α* can be attenuated, whereas the increased numbers of colocalizations of BrdU and DCX specific markers in the contused brain tissue can be enhanced by etanercept therapy during TBI. Thus, it appears that etanercept attenuates TBI in rats by reducing TNF-*α* contents and by enhancing newly formed neurogenesis in the contused brain tissues.

## Figures and Tables

**Table 1:** Antibodies used for immunofluorescence staining.

Antibody	Antigen	Host	Company	Catalog	Dilution
Primary antibody					

Anti-DCX antibody	Doublecortin (DCX, a marker for neuroblasts)	Rabbit	Cell Signaling Technology	4604	1 : 200
Anti-BrdU antibody	Bromodeoxyuridine (BrdU)	Rat	Abcam	ab6326	1 : 200

Secondary antibody (conjugation)					

Anti-rabbit IgG-H&L antibody (FITC)	Rabbit IgG	Goat	Abcam	ab6717	1 : 400
Anti-rat IgG antibody (DyLight 594)	Rat IgG	Goat	Abcam	ab96931	1 : 400
